# Xanthan- and Rice Cereal-Based Thickeners in Infants: A Multidisciplinary Single-Center Experience

**DOI:** 10.1097/PG9.0000000000000190

**Published:** 2022-04-08

**Authors:** Andrea Abdulezer, Patrick Mooney, Marie-Eve Besner, Sophie Laniel, Sarah Milton, Christine Labelle, Guilherme M. Sant’Anna, Ana Sant’Anna

**Affiliations:** From the * University of Montreal, Montreal, Quebec, Canada; † McGill University, Montreal, Quebec, Canada; ‡ Montreal Children’s Hospital, McGill University Health Center, Montreal, Quebec, Canada; § Montreal Children’s Hospital, McGill University Health Center, Montreal, Quebec, Canada; ‖ Neonatal Division Montreal Children’s Hospital McGill University Health Center, Montreal, Quebec, Canada; ¶ Division of Pediatric Gastroenterology, Montreal Children’s Hospital, McGill University Health Center, Montreal, Quebec, Canada.

**Keywords:** infants, milk thickeners, rice cereal-based thickener, video fluoroscopy swallowing study, Xanthan-based thickener

## Abstract

Some infants aspirate thin liquids and must be fed thickened liquids in order to protect the lungs. However, thickeners have not been fully studied for safety. Xanthan-based thickeners have been implicated in the development of necrotizing enterocolitis and rice cereal-based thickeners have been associated with constipation and excessive weight gain. The aim of this study was to compare rates of adverse events between both thickeners.

**Methods:**

Single-center retrospective chart review conducted at a tertiary pediatric care center between January 2013 and July 2017. All infants deemed unsafe for oral feeding and treated with xanthan- or rice cereal-based milk thickeners were included. Data were extracted from the medical records and patients categorized according to the type of thickener. Primary outcome was the occurrence of diarrhea, constipation, overweight, and obesity at 3–6 and 6–12 months after thickener initiation. Appropriate statistical tests were used. In addition, an e-mail was sent to 14 level III Canadian Pediatric hospitals inquiring about their practice.

**Results:**

We identified 53 patients to be included in the study; 20 used xanthan-based- and 33 used rice cereal-based milk thickeners. Rates of diarrhea, constipation, overweight, and obesity at 3–6 and 6–12 months after initiation were not different between thickeners. Important variability concerning thickening practices was reported by the 8 centers that responded.

**Conclusions:**

In infants treated with milk thickeners, xanthan-based or rice cereal-based thickeners may have similar safety profiles that require further investigation including a larger number of patients.

**What Is Known**

- Introduction of oral feeds has been associated with risks such as lung aspiration in high-risk infants.
- In these cases, thickeners are used but only a few safety studies were performed, and none compared xanthan- with rice cereal-based thickeners.

**What Is New**

- In a single-center cohort study, rates of diarrhea, constipation, overweight, and obesity were not different between xanthan- and rice cereal-based thickeners when assessed at 3–6 and 6–12 months after initiation.
- Important variability concerning thickening practices was reported by Canadian centers.

## INTRODUCTION

Prolonged enteral tube feeding in infants has been associated with complications including aspiration pneumonia, feeding intolerance, food refusal, and poor development of oromotor feeding skills (1–5). However, introduction of oral feedings has been related to risks of milk lung aspiration as evidenced clinically and/or by abnormal video fluoroscopy swallowing study (VFSS). In these cases, food thickeners are commonly used to safely initiate and progress with oral feedings. Unfortunately, to date, only a few studies (6–10) on the safety and efficacy of thickeners have been conducted in this age group.

Xanthan-based thickeners have stable viscosity with ease of use but have been implicated in the development of necrotizing enterocolitis (NEC) in premature infants (6–8). Rice cereal-based thickeners have been associated with constipation and excessive weight gain and are contraindicated in patients with food allergy (9,10). Xanthan-based thickeners provide adequate thickening of both breast milk and formula (11) but progressive thickening may occur over time (11,12). Rice cereal-based thickeners are unsuccessful in thickening breast milk and have been associated with inconsistent thickening of formula with lack of stability over time, resulting in a nonhomogeneous mixture (11,12). Concerns of adding thickeners to any formula also include increased fluid osmolality, diarrhea and dehydration, constipation, and overweight or obesity (6–10). Indeed, caution must be taken when thickening a calorically concentrated formula, which might surpass the safe osmolality range leading to potential clinical complications (13).

In Canada, a xanthan-based thickener is only recommended for children > 3 years of age, but a recent pilot survey of Canadian Pediatric Centers found that both xanthan- and rice cereal-based thickeners are commonly used in infants (14). Therefore, this study aimed to evaluate the incidence of adverse effects of xanthan- and rice cereal-based thickened feeds used in infants over a 1-year period. In addition, clinical outcomes were evaluated. Concomitantly to this analysis, a quick survey concerning milk thickening practices in Canadian pediatric centers was performed.

## MATERIALS AND METHODS

This single-center retrospective cohort study was conducted at the Montreal Children’s Hospital, a tertiary pediatric care center. Between January 2013 and July 2017, all infants at high risk for dysphagia, that is, deemed unsafe for per os feeding after an occupational therapy (OT) evaluation, with a failed VFSS, and started on oral feeding with xanthan-based or rice cereal-based milk thickeners, were identified and included. There were no exclusion criteria. Data were extracted from the medical records. Patients were categorized according to the type of thickener used: xanthan-based or rice cereal-based. In May 2014, a new protocol for milk thickening was developed and implemented at the Montreal Children’s Hospital, recommending the rice cereal-based thickener as the first choice based reports of increased incidence of NEC with xanthan-based cereal (6–8). The study was approved by the institution’s research ethics board.

Baseline variables before initiation of thickeners were recorded close to the time of initial failed VFSS (see definition below). The following patient demographics were collected: gender, gestational age (GA), corrected gestational age, primary diagnosis, VFSS grade, and type of milk used. Achievement of full oral feeds or need of nasogastric or gastrostomy feeds was recorded after 3–6 and 6–12 months of thickener use. Additionally, the following anthropometric parameters were retrieved from the medical records at baseline and at 3–6 and 6–12 months after initiation of the thickener: weight, length, and head circumference. The weight-for-age, length-for-age, weight/length ratio, head circumference-for-age, and *z* scores were calculated. Moreover, a change in weight *z* score from baseline to 3–6 and 6–12 months after initiation of the thickener was calculated.

The primary outcome was the occurrence of diarrhea, constipation, overweight, and/or obesity at 3–6 and 6–12 months after thickener initiation. Patients were considered overweight or obese if weight-for-length were above the 97th or 99.9th percentile, respectively (15,16). Diarrhea was defined as an increase in the number of stools or the presence of looser stools more than is normal for the individual (> 3 bowel movements each day). Constipation was defined as a delay or difficulty in passage of bowel movements present for 2 or more weeks, which caused the patient a significant distress. Other outcomes evaluated were food refusal, gastroesophageal reflux (GER), or NEC.

### Clinical Definitions

Corrected ages were calculated for premature infants (< 37 weeks) at all time points. Primary diagnosis at initiation of the thickener was divided into the following categories: prematurity, genetic, neurologic, otolaryngologic, respiratory, and feeding problems, and others. VFSS was graded using the following scale: (1) normal; (2) no aspiration, but not entirely normal (ie, pooling, penetration); (3) aspiration with some, but not all, consistencies; and (4) aspiration/unsafe for oral feeding with all consistencies (4). Food refusal was defined as clinically documented limitation in oral intake with or without oral hypersensitivity determined by OT assessment. GER was defined by the presence of clinical symptoms (regurgitation, vomiting, excessive eructation or hiccups, irritability while feeding) or treatment with proton pump blockers or H2 blockers.

In addition to this retrospective analysis, an e-mail was sent to 14 level III pediatric hospitals across Canada inquiring about their practice or protocol concerning the use of thickeners in infants. There was no specific questionnaire and centers were expected to provide any answer related to their practice in this specific subject.

### Statistical Analysis

Continuous variables are presented as n (%), mean ± SD, median (interquartile range), or range (minimum–maximum) and were analyzed using the Student *t* test for comparisons between groups: xanthan- versus rice cereal-based thickeners. Categorical variables were analyzed by using the Pearson chi-square or Fisher exact test. Statistics were computed using Stata (R2017a; MathWorks, Natick, MA), and *P* value of < 0.05 was considered significant. Due to the sample size of the study, a power analysis was performed.

## RESULTS

During the study period, 53 patients were identified and included in the study; 20 used xanthan-based thickeners and 33 were managed with rice cereal-based milk thickeners. The population characteristics are presented in Table 1. Infants treated with rice cereal-based thickeners were of lower GA at birth, had worse VFSS scores before initiation of the thickener and received more semi-nectar thickening consistencies.

**TABLE 1.** Population demographics

	Xanthan Based (n = 20)	Rice Based (n = 33)	*P*
Gestational age at birth, wk	38.4 (36.2–39.4)	38.2 (34.1–39.2)	0.033
Gender, male	12 (60)	13 (39)	0.145
Video fluoroscopy swallow study			
Corrected age at study (d)	60 (30–127.5)	45 (21–135)	0.205
Grade 2	12 (60)	5 (15)	0.003
Grade 3	7 (35)	23 (70)	
Grade 4	1 (5)	5 (15)	
Type of milk			
Breast milk	4 (20)	4 (12)	0.105
Formula	8 (40)	23 (70)	
Unknown	8 (40)	6 (18)	
Primary diagnosis			
Prematurity	—	2 (6)	0.298
Otorhinolaryngology	3 (15)	11 (34)	
Genetic	—	1 (3)	
Neurologic	3 (15)	2 (6)	
Respiratory	6 (30)	5 (15)	
Feeding problems	6 (30)	4 (12)	
Cardiac	2 (10)	8 (24)	
Thickness recommended			
Honey	2 (10)	1 (3)	0.009
Semi-nectar	—	9 (27)	
Nectar	18 (90)	23 (70)	

Results are presented as mean ± SD, median (interquartile range), or N (%).

In both groups, the number of patients receiving milk thickener decreased significantly during the first year: xanthan-based thickener—decreased to 14 patients after 3–6 months and only 2 patients after 6–12 months (*P* = 0.0002); rice cereal-based thickener—decreased to 23 patients after 3–6 months and 8 patients after 6–12 months (*P* = 0.0005). This was a result of improvement in the VFSS during the study period.

### Primary Outcome

There were no differences between xanthan- and rice cereal-based thickeners for any of the adverse events compared: diarrhea, constipation, overweight, and obesity at 3–6 and 6–12 months after initiation of the thickener (Table 2). However, due to the small sample size, the statistical power of the primary outcome was 16% for an alpha of 0.05. Interestingly, prolonged use of rice cereal thickener was significantly associated with increased rates of constipation. After 3–6 months, 5 of 23 (22%) of the patients receiving this thickener had constipation compared with 7 of 8 (87.5%) infants still on this thickener after 6–12 months (*P* = 0.002). The percent increase in constipation rates was also noted for prolonged use of xanthan-based thickener (from 36% to 100%) but results were not statistically significant (*P* = 0.466), likely due to the small number of patients still receiving this type of thickener after 6–12 months (n = 2). Anthropometric parameters at baseline, 3–6 and 6–12 months after the initiation of the thickeners were also compared between the 2 groups (Tables 3 and 4). No significant differences were noted except for length at 6–12 months, which was at a lower percentile in the group of infants treated with rice cereal thickeners.

**TABLE 2.** Feeding practices and adverse effects related to the thickeners

	Xanthan Based (n = 20)	Rice Based (n = 33)	*P*
After 3–6 mo of use			
Using thickener	n = 14 (70)	n = 23 (70)	1
Corrected age (mo)	9 (7.1–13.7)	8.3 (7.5–12.1)	0.280
Feeding			
Full per os	8 (86)	9 (39)	0.327
Nasogastric	6 (43)	13 (56)	0.507
Gastrostomy	—	1 (4)	1
Food refusal	6 (43)	5 (22)	0.267
Adverse effects*	6 (43)	6 (33)	0.471
Diarrhea	3 (21)	1 (4)	0.141
Constipation	5 (36)	5 (87)	0.453
Overweight	0	1 (4)	1
Obesity	1 (7)	1 (4)	1
Gastroesophageal reflux	6 (42)	18 (78)	0.039
After 6–12 mo of use			
Using thickener	n = 2 (10)	n = 8 (24)	0.286
Corrected age (mo)	14.0 (13–19.7)	14 (12.8–15.9)	0.324
Feeding			
Full per os	—	1 (12.5)	1
Nasogastric	—	3 (37.5)	1.00
Gastrostomy	2 (100)	4 (50)	0.466
Food refusal	2 (100)	7 (87.5)	1
Adverse effects*	1 (50)	7 (87.5)	0.377
Diarrhea	—	—	—
Constipation	1 (100)	7 (100)	1
Overweight	—	—	—
Obesity	—	—	—
Gastroesophageal reflux	2 (100)	8 (100)	1

Corrected age of assessment. Results are presented as median (interquartile range) and N (%).

*More than 1 adverse effect could be present.

**TABLE 3.** Initial anthropometric measurements

	Xanthan Based (n = 20)	Rice Based (n = 33)	*P*
Corrected age (d)	60 (31.5–144)	63 (20.5–127)	0.347
Weight (kg)	4.85 (3.9–7.2)	4.7 (3.45–5.95)	0.245
Length (cm)	56.2 (50–68.4), n = 15	53.6 (48.5–57.5), n = 22	0.023
Head circumference (cm)	37.2 (35.5–43.5), n = 14	35.5 (34.5–37.3), n = 15	0.103
Weight centile	19.5 (9–59.5)	24 (9.5–42)	0.383
Length centile	36.8 (3.8–61.9), n = 15	13.2 (3.4–43.8), n = 22	0.078
Weight/length centile	30.5 (5.4–57), n = 15	43 (12.5–67.7), n = 22	0.400
Head circumference centile	66.4 (9.1–89.1), n = 14	38 (4.6–69.8), n = 15	0.198
Weight *z* score	–0.55 (–1.48 to 0.36)	–0.88 (–1.79 to –0.18)	0.212
Length *z* score	0.04 (–1.77 to 0.35), n = 15	–1.30 (–2.23 to –0.1), n = 22	0.495
Head circumference *z* score	–0.49 (–1.54 to 1.31), n = 14	–0.30 (–1.64 to 0.72), n = 15	0.362

Results are presented as median (interquartile range), n of cases, or N (%).

**TABLE 4.** Anthropometric parameters between 3–6 months and 6–12 months after initiation of the thickener

	Xanthan Based (n = 20)	Rice Based (n = 33)	*P*
Thickener for 3–6 mo	n = 15	n = 26	
Corrected age (mo)	9 (7.1–13.7)	8.3 (7.5–12.1)	0.218
Weight (kg)	8.5 (7.2–9.9)	7.8 (7.2–8.5), n = 25	0.448
Length (cm)	69.5 (66.5–73.5), n = 10	68 (64–70.9), n = 19	0.211
Head circumference (cm)	43 (42.3–46.7), n = 3	43 (42.1–43.8), n = 12	0.369
Weight centile	43.1 (9.9–64.6)	34.3 (5.3–60.1), n = 25	0.400
Length centile	26.2 (7.4–50.8), n = 10	13.8 (1.2–51), n = 19	0.388
Weight/length centile	41.6 (25.8–62.6), n = 10	61.8 (13.6–76.8), n = 19	0.424
Head circumference centile	29.1 (0.7–33.7), n = 3	22.4 (6.5–51.9), n = 12	0.245
ΔWz from baseline	–0.35 ± 1.04, n = 15	–0.34 ± 1.22, n = 25	0.978
Thickener for 6–12 mo	n = 16	n = 24	
Corrected age (mo)	14.0 (13–19.7)	14 (12.8–15.9)	0.125
Weight (kg)	9.8 (8.3–10.9)	9 (8.2–9.7), n = 23	0.265
Length (cm)	78 (71–80.5), n = 13	75 (73.2–76.5), n = 17	0.050
Head circumference (cm)	45 (44.5–46.2), n = 7	44.5 (43.5–45.5), n = 10	0.095
Weight centile	33 (16.5–75)	11 (6–64.8), n = 23	0.169
Length centile	41 (5–67), n = 13	13 (1–47), n = 17	0.004
Weight/length centile	45 (10–63), n = 13	44 (15–64), n = 17	0.408
Head circumference centile	27 (13–60), n = 7	18 (1–40), n = 10	0.076
ΔWz from baseline	–0.24 ± 0.88, n = 16	–0.37 ± 1.5, n = 23	0.752

Results are presented as median (interquartile range), mean ± SD, or n (%). ΔWz = change in *z* score for weight.

There were no differences between thickeners on feeding practices and food refusal, GER, or NEC at 3–6 and 6–12 months after the initiation of the thickeners (Table 2).

Nine Canadian centers replied to the e-mail and 8 shared their thickening practices or protocols; 1 center stated no use of thickeners in infants. Appendix 1 (http://links.lww.com/PG9/A77) summarizes the population age, type of thickener recommended, criteria to use the thickener, and specific concerns of their respective practices. Five (55%) centers stated using milk thickeners in infants, whereas 3 centers specified using thickeners only in infants with > 42 or 44 weeks of corrected GA. Those centers never or rarely use thickeners in infants younger than this corrected gestational age. Interestingly, 7 different types of thickeners were reported. The most commonly used was rice cereal (50%), followed by carob bean gum (Gelmix) and Enfamil A+ (38%). Two centers (25%) use xanthan-based thickener and 4 (50%) centers use rice cereal-based thickeners. The most common indication for use was risk of pulmonary aspiration (89%). Only 4 centers reported concerns about adverse effects of milk thickeners: issues related to tolerance (2), constipation or excessive weight gain (1), and NEC (1).

## DISCUSSION

In this retrospective cohort study, xanthan-based and rice cereal-based milk thickeners were compared in infants that have failed initial OT assessment and VFSS. No statistical differences, but with a low statistical power, were noted between thickeners for diarrhea, constipation, overweight, and obesity at 3–6 and 6–12 months after initiation. An increased rate of constipation was observed in infants receiving rice cereal for a prolonged time period (6–12 months).

Advancements in neonatal medicine have significantly increased survival rates of infants born prematurely with low birth weight or complex medical issues (17). This has been associated with an increased prevalence of neonatal and pediatric feeding and swallowing disorders, including poor sucking/swallow/breathing coordination, oral motor delays, dysphagia, food refusal, GER, malnutrition, and delayed transition to appropriate textures or foods (17). Therefore, safe introduction of oral feeds in some of these infants may require the use of thickening agents. However, to date, few studies have assessed the safety of different types of thickeners in this population.

Concerns over the relationship between xanthan-based thickeners and development of NEC (6–8) have led to the adaptation of thickening protocols at many centers worldwide. Rice cereal-based thickeners became the most common thickening agent, as demonstrated in the response of the 8 Canadian centers. However, some concerns have been raised with the use of rice cereal-based thickener, given its association with increased rates of constipation and excessive weight gain (9). In the present study, 33 infants were treated with rice cereal-based thickener without any differences in adverse effects when compared with xanthan-based thickener. Indeed, rates of diarrhea, overweight, and obesity were very low with both groups. The most common adverse event was constipation that increased significantly with the prolonged use of rice cereal-based thickener (6–12 months) but was not different from xanthan-based thickener. This is reassuring as the use of thickeners significantly decreased over time, with a very small percentage of infants still requiring thickeners between 6 and 12 months after initiation. According to the chart review, this decrease with age seemed to be related to improvement of the clinical status of the baby as the VFSS normalized. However, improvement in per os tolerance or discontinuation based on parents decision cannot be excluded. Mascarenhas et al (18) have reported a rate of constipation of only 20% in infants receiving rice cereal for thickening. Our data show a decrease in the use milk thickener over the first year of life. This decrease with age seems to be related to improvement of the clinical status of the baby as the VFSS normalized. However, improvement in oral tolerance or discontinuation based on parents decision cannot be excluded. Finally, the type of thickener used did not affect growth, as no clinically relevant differences in anthropometric measures were apparent between xanthan- and rice cereal-based thickeners from baseline to 3–6 and 3–6 months of treatment. Of note, no patient developed NEC.

A high variability in clinical practice concerning thickening of milk was reported by the Canadian centers. Protocols and practices for thickener use in infants less than 1 year old differed and 7 different thickening products are used among these centers (Appendix 1, http://links.lww.com/PG9/A77). Such practice variability is likely due to the low level of evidence towards the use of a specific type of thickener in these cases.

This study has several limitations. As any retrospective analysis, some data are missing, mostly related to measurements of length and head circumference over time. The data collection regarding reflux was based on a chart review and not necessarily is accurate, and this needs to be considered in the results. Although prescribed, we could not infer the true adherence to thickening protocols outside the hospital setting. Finally, as the food diary of the infants was not part of this project, it was assumed that the constipation observed was associated with the use of thickeners, as it is a known side effect of rice cereal. For the small difference noted between the 2 types of thickeners in the primary outcome, a type II error cannot be excluded due to the low calculate power and a larger number of patients would be needed to provide a power of 80% using an alpha of 0.05.

However, the study has important strengths. Relevant and unique data were obtained from 53 high-risk patients started on thickening at < 1 year of age after failing initial OT assessment and VFSS. These patients were followed prospectively in an academic institution for a period of 12–18 months after thickening initiation. Given the increased use of rice cereal thickening and paucity of data on the use of these products in this young and high-risk population, the study provides baseline information for calculation of sample sizes needed in future studies. Other types of thickening practices are available but might not be suited for all patients due to the cost, limited accessibility, and need of precise steps to prepare thicken liquids. Nevertheless, more information on safety and efficacy of these products is also warranted.

## ACKNOWLEDGMENTS

G.M.S.A. and A.S.A. contributed to the conception and design of the research. A.A. and P.M. contributed to the acquisition of the data. G.M.S.A. contributed to the analysis of the data. M.-E.B., S.L., S.M., C.L., G.M.S.A., and A.S.A. contributed to the interpretation of the data. A.A., G.M.S.A., and A.S.A. drafted the article. All authors critically revised the article, agree to be fully accountable for ensuring the integrity and accuracy of the work, and read and approved the final article.

## Supplementary Material


