# Supplementary material for: Xanthan- and Rice Cereal-Based Thickeners in Infants: A Multidisciplinary Single-Center Experience
Source: JPGN Rep. 2022 Apr 8;3(2):e190. doi: 10.1097/PG9.0000000000000190 (PMC10158338; doi:10.1097/PG9.0000000000000190)
Supplement: Supplementary file 1 [file pg9-3-e190-s001.pdf]

Supplemental Table. Canadian Pediatric Centers Thickening Practices for Infants < 1 year old

Center	Population	Thickener Recommended	Criteria for Use	Concerns
A	< 1 year old	Rice cereal & Gelmix®	Infants at risk of aspiration	N/A
B	< 1 year old	*Option 1* - Enfamil A+ for feeding babies who frequently spit up<br><br>*Option 2* - Rice cereal	Infants at risk of aspiration or experiencing swallowing difficulties<br><br>If Enfamil A+ not tolerated at normal concentration and if the infant does not require enriched calories	Thickness Level 1 IDDSI at normal concentration not always tolerated by the patient<br><br>Thickness Level 2 IDDSI mixed at higher concentration is better tolerated however not suited for all infants<br><br>Constipation, Excess weight gain
C	< 1 year old	Resource ThickenUp®	Infants at risk of aspiration or experiencing swallowing difficulties	-
D	< 1 year old	Resource ThickenUp Clear®	Infants at risk of aspiration or experiencing swallowing difficulties who failed Dr. Brown's nipples or do not fit criteria to try it	Risk of NEC related to using a Xanthan-based thickener – parents must sign a waiver before thickener can be used
E	< 1 year old and > 42 weeks gestational age	Rice cereal & Gelmix®	Infants at risk of aspiration	-
F	< 1 year old and > 44 weeks gestational age	*Option 1* - Positioning and mechanical methods<br><br>*Option 2* - Enfamil A+ for feeding babies who frequently spit up	Infants at risk of aspiration<br><br>Infants at risk of aspiration	Thickness at 68 kcal/100 ml reaches "slightly thickened" consistency and may not be sufficiently tolerated<br><br>Thickness at 81 kcal/100 ml reached "mildly/nectar thick" consistency and may not be sufficiently tolerated
G	Preterm infants < 42 weeks	*Option 1* - No thickener used	Infants at risk of aspiration	-

		Option 2 - Rice Cereal thickened formula (rare)		
	Preterm infants > 42 weeks + Term infants < 4 months	*Option 1* - Breast milk+ Gelmix®<br><br>*Option 2* - Rice cereal thickened formula (15 ml rice cereal + 60 ml formula)<br><br>*Option 3* - Gelmix® thickened formula	Infants at risk of aspiration<br><br>Infants at risk of aspiration<br><br>Infants at risk of aspiration<br>If financially possible for the family	-<br><br>Remove infant from thickener as soon as possible<br><br>-
	Term infants > 4 months old < 1 year old	*Option 1* - Rice cereal thickened formula<br><br>*Option 2* - Formula thickened with Quick Thick®	Infants at risk of aspiration<br><br>Infants at risk of aspiration	-<br><br>-
H	< 1 year old	*Option 1* - Pectin Gel®<br><br>*Option 2* - Enfamil A+ for feeding babies who frequently spit up	NICU patients with short gut<br><br>Patents with gastric reflux	-

Legend: IDDSI = International Dysphagia Diet Standardisation Initiative
(https://iddsi.org/IDDSI/media/images/Complete_IDDSI_Framework_Final_31July2019.pdf)
